# Strutting Through: Migration of Fractured Inferior Vena Cava Filter Through the Right Ventricle

**DOI:** 10.1016/j.atssr.2022.10.001

**Published:** 2022-10-12

**Authors:** Nader Sarkis, Keith B. Allen, Karthick Vamanan

**Affiliations:** 1 Department of Cardiothoracic and Vascular Surgery, Saint Luke's Mid America Heart Institute, Kansas City, Missouri

## Abstract

A 68-year-old woman who had a retrievable inferior vena cava filter implanted 10 years ago presented with sudden-onset back pain. Initial computed tomography angiography demonstrated migration of a fractured strut that appeared embedded in the anterior right ventricular free wall without pericardial effusion. Subsequent gated computed tomography of the chest demonstrated further migration of the fragment, which was now penetrating the right ventricular free wall and extending into the pericardial sac. At the time of operative removal through a limited left anterior thoracotomy, the fractured strut had migrated completely through the heart and was positioned posteriorly in the pericardial sac.

The use of retrievable inferior vena cava (IVC) filters has increased as a means to provide temporary protection from a potential pulmonary embolism, usually in the setting of urgent or emergent operation, when interruption of anticoagulation will occur. Whereas complications from permanent IVC filters, such as erosion, perforation, and migration, are infrequent, retrievable filters are less robust than their permanent counterparts to facilitate removal and therefore are prone to fracture if they are not removed 3 to 6 months after placement. Unfortunately, retrievable filters are often not removed in a timely fashion in as many as 60% of patients.^1^ We present a case of a retrievable IVC filter strut fracture with migration through the right ventricular free wall and into the pericardial sac that is documented during the course of 12 hours. The strut was successfully removed through a limited left anterolateral thoracotomy with pericardiotomy followed by staged removal of the IVC filter 3 months later.

A 68-year-old woman with a history of hypertension presented to an emergency department with 24 hours of back pain that began abruptly and woke her from her sleep. The patient’s past medical history was pertinent for Crohn disease that required a colon resection and colostomy 8 years ago complicated by a deep vein thrombosis and pulmonary embolism requiring warfarin. At the time of colostomy takedown, a Recovery G2 (Bard) retrievable IVC filter was implanted. She recovered uneventfully and discontinued her anticoagulation; however, her IVC filter was never removed. Her pain was described as waxing and waning with only partial control with over-the-counter medications. Concern for dissection prompted nongated computed tomography (CT) angiography of the chest, abdomen, and pelvis, which showed no evidence of dissection. However, the radiologist did note a metallic fragment embedded in the anterior right ventricular free wall without a pericardial effusion (Figure A) and an IVC filter that had only 11 struts when there should be 12 (Figure B).

**Figure:**
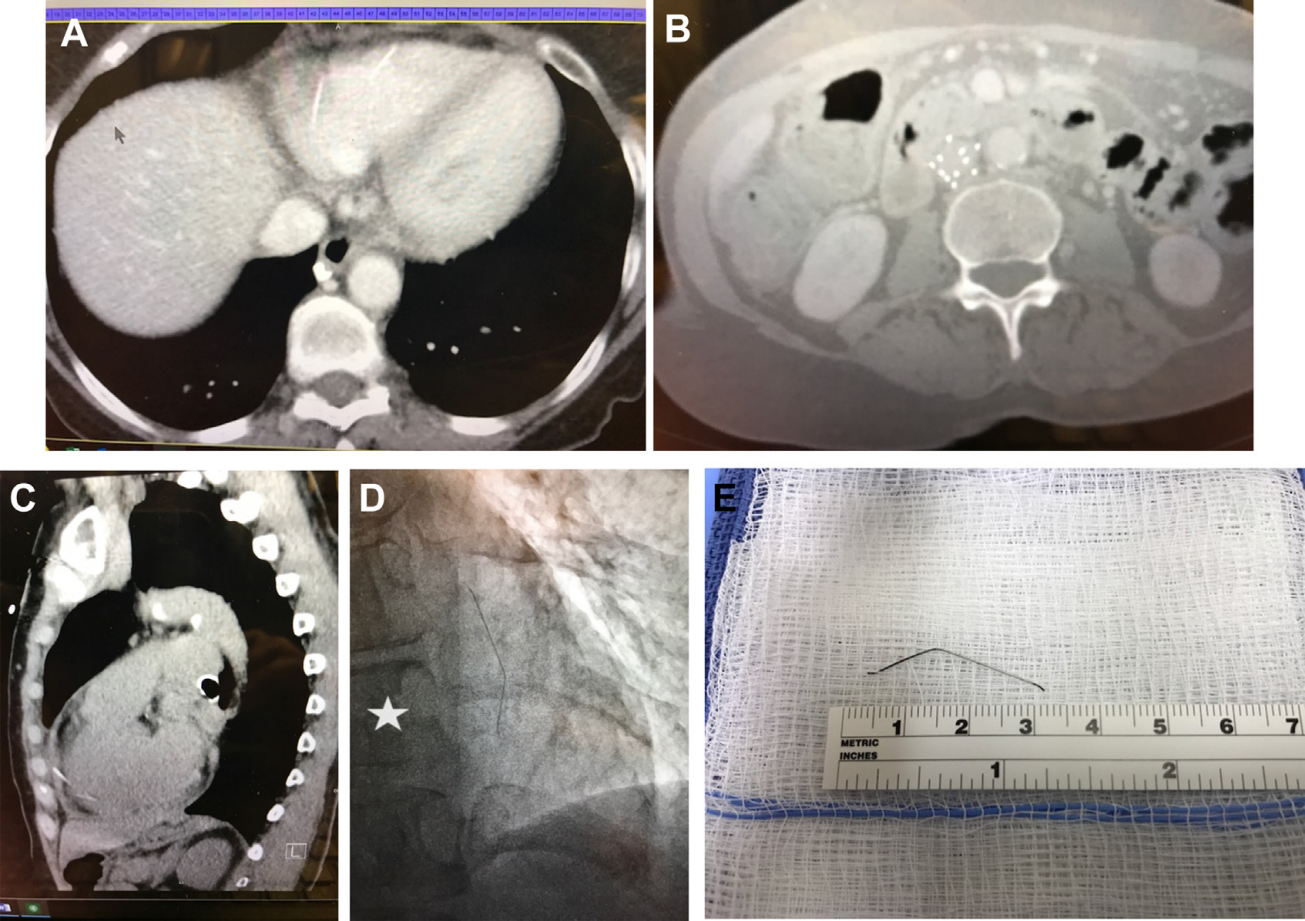
Computed tomography angiography of the chest and abdomen demonstrates (A) a metallic fragment (arrow) embedded in the anterior right ventricular free wall without a pericardial effusion and (B) an inferior vena cava filter that had only 11 struts when there should be 12. (C) Subsequent gated computed tomography angiography of the chest demonstrates further migration of the fractured strut, which is now penetrating the right ventricular free wall and extending into the pericardial sac with a new small effusion. (D) At the time of operation, a lateral chest radiograph demonstrated further migration of the strut into the posterior pericardium parallel to the spine (star). (E) After a limited left anterior lateral thoracotomy and pericardiotomy, the fractured strut was removed uneventfully.

She was transferred to our facility, where on arrival she characterized her pain as more intense and different in location. Gated CT angiography of the chest was performed; it now demonstrated that the fragment was penetrating the right ventricular free wall and extending into the pericardial sac, which now had a new small effusion (Figure C). Operative removal was planned, but on arrival to the hybrid room, her pain was significantly better and fluoroscopy was unable to locate the metallic fragment near its last known location by CT. Because further migration was suspected, a lateral chest radiograph (Figure D) was obtained in the operating room, which demonstrated that the strut was now parallel to the spine and likely in the posterior pericardial sac.

A limited left anterior lateral thoracotomy was performed, which identified a pericardial sac distended with blood. The pericardium was incised and drained, and a narrow malleable retractor was used to retract the heart, allowing visualization and removal of the fractured strut (Figure E) from the posterior pericardial sac. The perforation site was never visualized; however, no bleeding was noted, and the incision was closed. The patient made an uneventful recovery and was discharged on postoperative day 2. After a full recovery, the IVC filter was removed uneventfully by the vascular surgeon through a right internal jugular approach.

## Comment

IVC filters, particularly retrievable IVC filters, are increasingly used in the management of patients with deep vein thrombosis in whom anticoagulation is contraindicated or whose anticoagulation must be interrupted for urgent or emergent operation.^2^ Although clear indications for the use of IVC filters are outlined by the American College of Chest Physicians,^3^ as many as 40% of patients may receive IVC filters outside guideline recommendations.^4^ A reported complication rate of 1% to 2% with use of permanent IVC filters led to the development of retrievable filters in the hope of reducing complications such as perforation, migration, and fracture.^5^ To facilitate removal, retrievable filters are constructed less robust than permanent filters; this design feature unfortunately makes them more vulnerable to complications such as fracture if they are not removed in a timely fashion when they are no longer needed. In fact, in some reports, <40% of retrievable IVC filters are ever actually removed.^1^

Regarding the observed time between the IVC filter placement and embolization, the literature is inconclusive. Hussain and coworkers^6^ described a case of embolization and cardiac tamponade only 4 hours after filter implantation.

Management of embolized IVC filter fragments must be individualized on the basis of size and shape of the fragment and the potential complexity of surgical or endovascular removal. Embolization of fractured IVC filter struts to the lung has been reported for which conservative management has been successfully used.^7^^,^^8^ This case describes an embolized strut to the heart where the migration path has been documented with sequential imaging. It highlights the rapidity with which IVC filter struts can migrate and for which the surgical approach for removal may be altered according to where the fragment is at the time of surgical removal.
